# Supplementary material for: A Saliva-Based RNA Extraction-Free Workflow Integrated With Cas13a for SARS-CoV-2 Detection
Source: Front Cell Infect Microbiol. 2021 Mar 16;11:632646. doi: 10.3389/fcimb.2021.632646 (PMC8009180; doi:10.3389/fcimb.2021.632646)
Supplement: Supplementary Figure 1 — Standardization of various primers for the detection of SARS-CoV-2. (A) Detection of S gene amplicon by performing RT-qPCR using the S-P-1 primers. The desired amplicon size was obtained under all the annealing temperatures tested (112bp). S gene plasmid was used as template. (B) Similarly, S-P-4 primer for S gene amplification was used (134bp). (C) CDC-approved N-1 primer was used for PCR on N gene plasmid with a single amplicon (72bp) (D) PCR was performed using N-P1 primer which produced multiple amplicons. Black arrow head indicates the predicted amplicon size. The sequence of the respective primers is given in Supplementary Table 1. [file Presentation_1.pptx]

## Slide 1
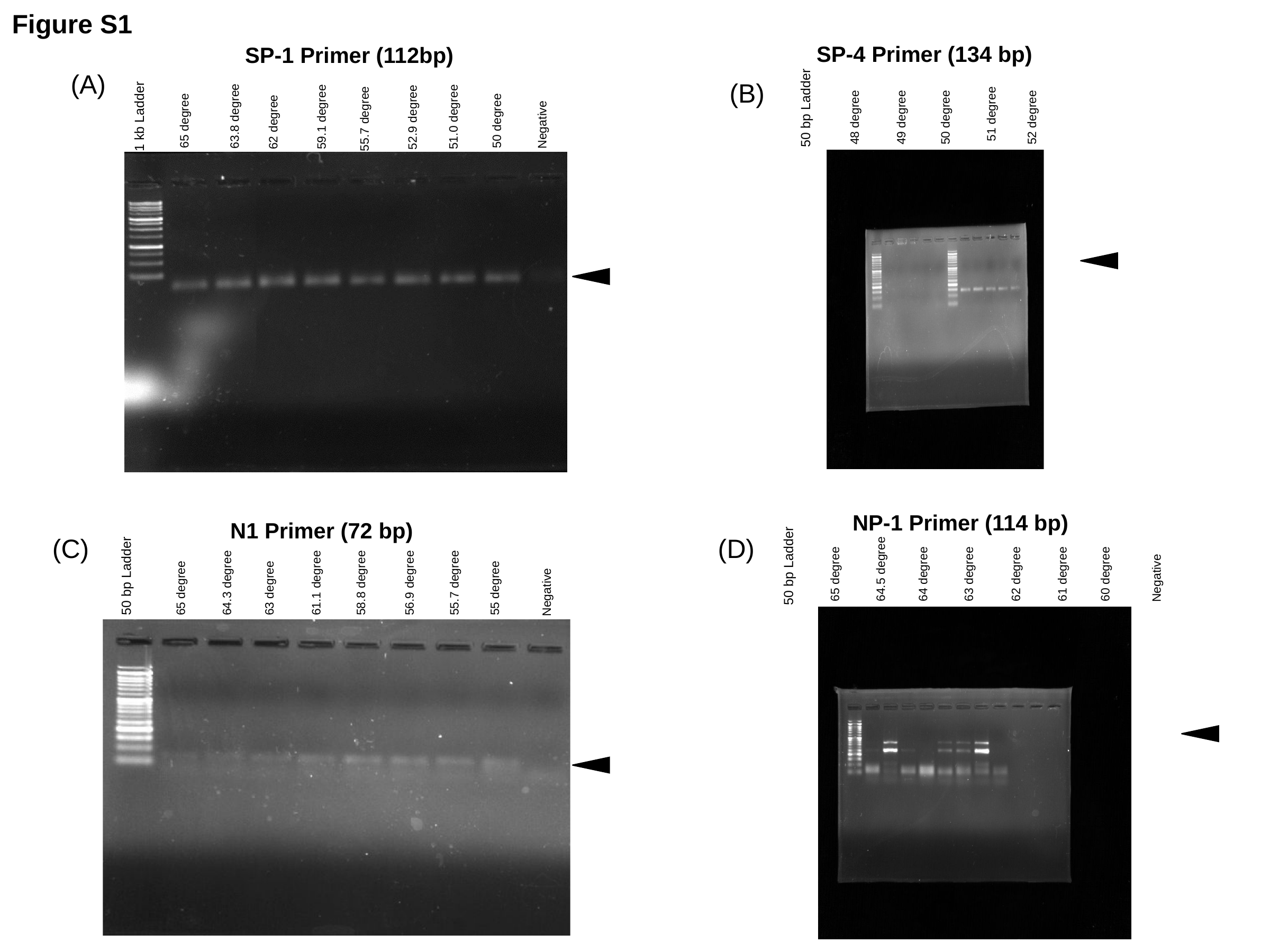

Figure S1
63.8 degree
51.0 degree
59.1 degree
52.9 degree
55.7 degree
50 degree
65 degree
62 degree
1 kb Ladder
Negative
48 degree
49 degree
50 degree
 51 degree
52 degree
50 bp Ladder
SP-4 Primer (134 bp)
SP-1 Primer (112bp)
(A)
(B)
64.5 degree
65 degree
64 degree
63 degree
62 degree
61 degree
60 degree
50 bp Ladder
Negative
50 bp Ladder
Negative
55.7 degree
64.3 degree
58.8 degree
61.1 degree
56.9 degree
65 degree
63 degree
55 degree
NP-1 Primer (114 bp)
N1 Primer (72 bp)
(C)
(D)
