# Supplementary material for: A Saliva-Based RNA Extraction-Free Workflow Integrated With Cas13a for SARS-CoV-2 Detection
Source: Front Cell Infect Microbiol. 2021 Mar 16;11:632646. doi: 10.3389/fcimb.2021.632646 (PMC8009180; doi:10.3389/fcimb.2021.632646)
Supplement: Supplementary Figure 2 — SHERLOCK validation of saliva samples using later-flow strips and signal quantitation method. (A1) Paper-strip images of SARS-CoV-2 positive (left) and SARS-CoV-2 negative (right) samples which were subjected to SHERLOCK and LFA. Corresponding Ct values derived from RT-qPCR is shown above the Lateral-flow strip. The patient ID and Ct values are same as shown in Figure 2f. (A2) Corresponding T/C ratio of the images is shown. Red arrow head indicates the threshold T/C ratio (B1) Longitudinal test results of two patient saliva samples taken four times before the test results came negative. (B2) T/C ratio of the corresponding LFA images. [file Presentation_2.pptx]

## Slide 1
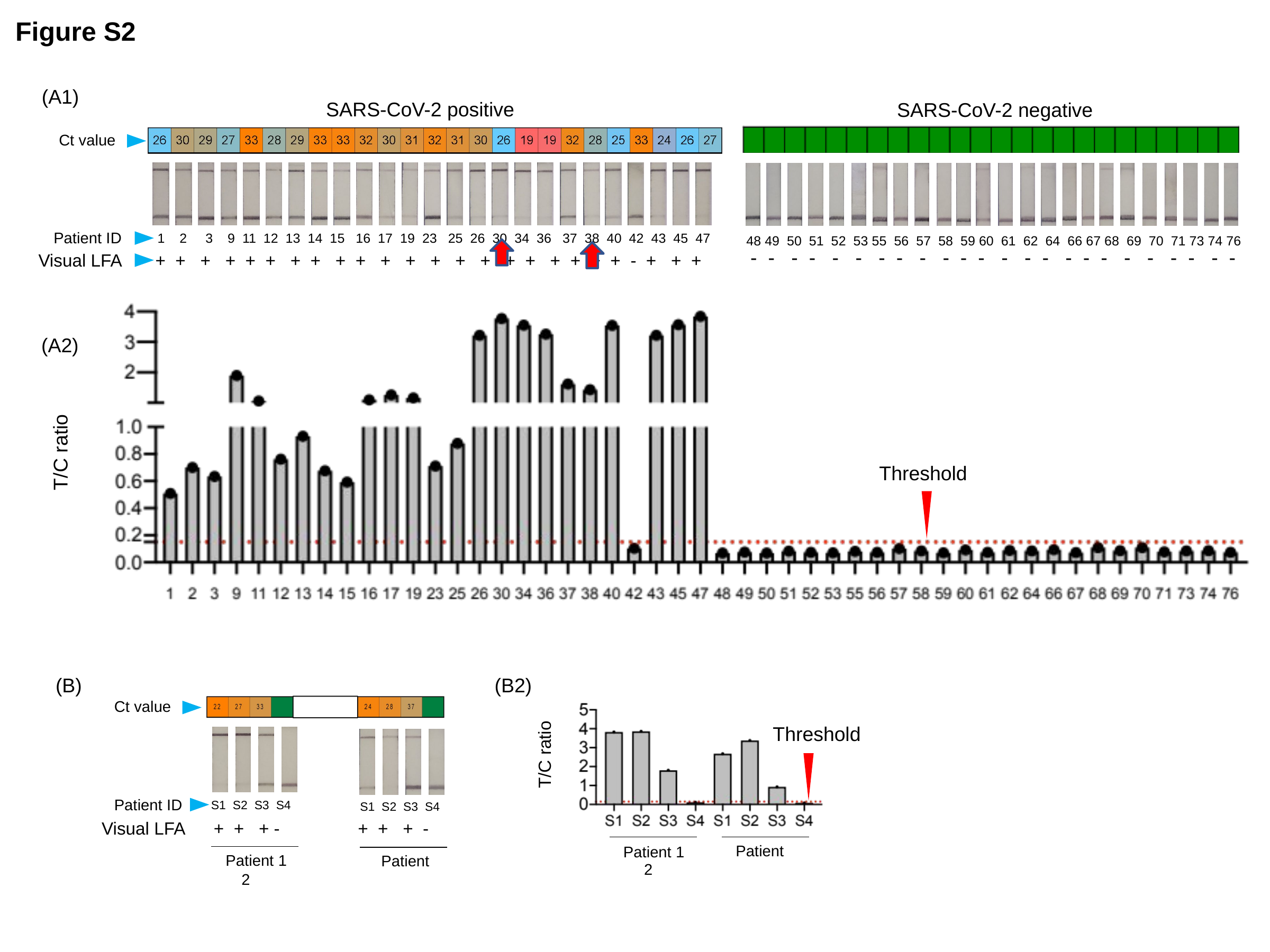

Figure S2
(A1)
SARS-CoV-2 positive
SARS-CoV-2 negative
Ct value
Patient ID
1 2 3 9 11 12 13 14 15 16 17 19 23 25 26 30 34 36 37 38 40 42 43 45 47
48 49 50 51 52 53 55 56 57 58 59 60 61 62 64 66 67 68 69 70 71 73 74 76
- - - - - - - - - - - - - - - - - - - - - - - -
Visual LFA + + + + + + + + + + + + + + + + + + + + + - + + +
(A2)
T/C ratio
Threshold
(B)
(B2)
Ct value
T/C ratio
 Patient 2
Patient 1
Threshold
Patient ID
S1 S2 S3 S4
S1 S2 S3 S4
Visual LFA + + + - + + + -
 Patient 1
 Patient 2
