# Supplementary material for: A Saliva-Based RNA Extraction-Free Workflow Integrated With Cas13a for SARS-CoV-2 Detection
Source: Front Cell Infect Microbiol. 2021 Mar 16;11:632646. doi: 10.3389/fcimb.2021.632646 (PMC8009180; doi:10.3389/fcimb.2021.632646)
Supplement: Supplementary file 5 [file Table_1.doc]

**Table S**1: List of primers, probes, crRNA, and synthetic DNA fragments

| **Name** | **Sequence** | | | **Sources** | | | **Note** | | **REF** |
| --- | --- | --- | --- | --- | --- | --- | --- | --- | --- |
| **RPA-and IVT** | | | | | | | | | |
| T7 S-gene  Fw | 5’**gaaattaatacgactcactataggg**AGGTTTCAAACTTTACTTGCTT TACATAGA-3’ | | | ILS | | | Lower‐case letters indicate overhang T7 promotor sequence | | Zhang Lab protocols, MIT |
| T7 S-gene  Rev | 5’-TCCTAGGTTGAAGATAACCCACATAATAAG-3’ | | | ILS | | |  | | Zhang Lab protocols, MIT |
| T7-Orf1ab  Fw | 5’**gaaattaatacgactcactataggg**CGAAGTTGTAGGAGACATTAT ACTTAAACC-3’ | | | ILS | | | Lower‐case letters indicate overhang T7 promotor sequence | | Zhang Lab protocols, MIT |
| T7-Orf1ab  Rev | 5’-TAGTAAGACTAGAATTGTCTACATAAGCAGC-3’ | | | ILS | | |  | | Zhang Lab protocols, MIT |
| T7-S-FP | **5’-gaaattaatacgactcactataggg**TTTTTCGGCTT-3’ | | | Sigma | | | Lower‐case letters indicate overhang T7 promotor sequence | | This study |
| T7-S-RP | 5’TCTAACAATAGATTCTGTTGGTTGGACTCTAAAGTT-3’ | | |  | | |  | | This study |
| T7-Orf1ab  FP | 5’-gaaattaatacgactcactatagggAATGGATAAT-3’ | | | Sigma | | |  | | This study |
| T7-Orf1a  RP | 5’-TTAGCTATAGTATCCCAAGGGACACTATTAACA-3’ | | |  | | |  | | This study |
| **crRNA** | | | | | | | | | |
| S gene | 5’**gauuuagacuaccccaaaaacgaaggggacuaaaac**GCAGCACCAGCUGUCCAACCUGAAGAAG-3’ | | | Synthego | | | Lower‐case letters indicate scaffold sequence | | Zhang Lab protocols, MIT |
| Orf1ab | 5’**gauuuagacuaccccaaaaacgaaggggacuaaaacc**CAACCUCUUCUGUAAUUUUUAAACUAU-3’ | | | Synthego | | | Lower‐case letters indicate scaffold sequence | | Zhang Lab protocols, MIT |
| **Reporter** | | | | | | | | | |
| Cas13a Reporter | 5’/56FAM/mArArUrGrGrCmAmArArUrGrGrCmA/3Bio/-3’ | | | IDT | | | Lateral Flow Reporter | |  |
| **Primer sequences** | | | | | | | | | |
| SP-1 (FP) | | | AGGTTTCAAACTTTACTTGCTTTACATAGA |  | | Used for PCR | | This study | |
| SP-1 (RP) | | | CTTATTATGTGGGTTATCTTCAACCTAGGA |  | | Used for PCR | |  | |
| SP-2 (FP) | | | ACTGTGCACTTGACCCTCTCT | Bio serve | | Used for PCR | | This study | |
| SP-2 (RP) | | | CACCAAAAGGGCACAAGTTTGTAA | Bio serve | | Used for PCR | | This study | |
| SP-3 (FP) | | | TGACCCTCTCTCAGAAACAAAGTGT | Bio serve | | Used for PCR | | This study | |
| SP-3 (RP) | | | AACTTCACCAAAAGGGCACAAGT | Bio serve | | Used for PCR | | This study | |
| SP-4 (FP) | | | 5’-ACTGTGCACTTGACCCTCTCTCAG-3’ | ILS | | Used for PCR | | This study | |
| SP-4 (FP) | | | 5’-AGTTTGTAATATTAGGAAATCTA-3’ | ILS | | Used for PCR | | This study | |
| NP-1 (FP) | | | 5’-ACCCGCAATCCTGCTAACAA-3’ | Bio serve | | Used for PCR | | This study | |
| NP-1 (RP) | | | 5’-ACGAGAAGAGGCTTGACTGC-3’ | Bio serve | | Used for PCR | | This study | |
| NP-2 (FP) | | | 5’-ATCACATTGGCACCCGCAAT-3’ | Bio serve | | Used for PCR | | This study | |
| NP-2 (RP) | | | 5’-GTTGCGACTACGTGATGAGGA-3’ | Bio serve | | Used for PCR | | This study | |
| NP-3 (FP) | | | 5-’CACATTGGCACCCGCAATC-3’ | ILS | | Used for PCR | | This study | |
| NP3 (RP) | | | 5’-GAGGAACGAGAAGAGGCTTG-3’ | ILS | | Used for PCR | | This study | |
| N1 (FP) | | | 5’-GACCCCAAAATCAGCGAAAT-3’ | Sigma | | Used for PCR | | Chantal et al., 2020 | |
| N1 (RP) | | | 5’-TCTGGTTACTGCCAGTTGAATCTG-3’ | Sigma | | Used for PCR | | Vogels et al., 2020 | |
| Orf1ab-FP | | | 5’-AGGAGACATTATACTTAAACCAGCA-3’ | Sigma | | Used for PCR | | This study | |
| Orf1ab-RP | | | 5’-TAGATCTGTGTGGCCAACCTC-3’ | Sigma | | Used for PCR | | This study | |
| Orf1ab-V-FP | | | 5’-CCCTGTGGGTTTTACACTTAA-3’ | Sigma | | Used for PCR | | This study | |
| Orf1ab-V-RP | | | 5’-ACGATTGTGCATCAGCTGA-3’ | Sigma | | Used for PCR | | This study | |
| RNase FP | | | 5’-AGATTTGGACCTGCGAGCG-3’ | Sigma | | Used for PCR | |  | |
| RNase RP | | | 5’-GAGCGGCTGTCTCCACAAGT-3’ | Sigma | | Used for PCR | |  | |
| **PROBES** | | | | | | | | | |
| SP-1  Probe sequence | | FAM-CTCCTGGTGATTCTTCTTCAGG BBQ | | Sigma | Used for PCR | | |  | |
| SP-1, SP-2, SP-3  Probe sequence | | 6FAM-AATCTATCAAACTTCTAACTTTA- BBQ | | ILS | Used for PCR | | | This study | |
| NP-1, NP-2, and NP3  Probe Sequence | | 6FAM-ACTTCCTCAAGGAACAACATTGCCA-BBQ | | ILS | Used for PCR | | | This study | |
| N1 Probe sequence | | FAM-ACCCCGCATTACGTTTGGTGGACC- BBQ | | Sigma | Used for PCR | | | Vogels et al., 2020 | |
| RNAse P | | FAM-TTCTGACCTGAAGGCTCTGCGCG- BBQ | | Sigma | Used for PCR | | | Vogels et al., 2020 | |

| **Template sequence for IVT** | | | | |
| --- | --- | --- | --- | --- |
| S-synthetic fragment 1 | **gaaattaatacgactcactataggg**AGGTTTCAAACTTTACTTGCTTTACATAGAAGTTATTTGACTCCTGGTGATTCTTCTTCAGGTTGGACAGCTGGTGCTGCAGCTTATTATGTGGGTTATCTTCAACCTAGGACTT | Xcelris | Used for IVT | This study |
| S synthetic fragment 2 | **gaaattaatacgactcactataggg**ACTGTGCACTTGACCCTCTCTCAGAAACAAAGTGTACGTTGAAATCCTTCACTGTAGAAAAAGGAATCTATCAAACTTCTAACTTTAGAGTCCAACCAACAGAATCTATTGTTAGATTTCCTAATATTACAAACTTGTGCCCTTTTGGTGAAGTT | Xcelris | Used for IVT | This study |
| Orf1ab  synthetic fragment | **gaaattaatacgactcactataggg**CTACCGAAGTTGTAGGAGACATTATACTTAAACCAGCAAATAATAGTTTAAAAATTACAGAAGAGGTTGGCCACACAGATCTAATGGCTGCTTATGTAGACAATTCTAGTCTTACTATTAA | Xcelris | Used for IVT | This study |

Abbreviations: FP: Forward Primer; RP: Reverse primer; Fw: Forward sequence; Rev: Reverse sequence. SP: S gene primer; NP: N gene primer; N1: USA CDC approved primer for N gene; RPA: Recombinase polymerase amplification; IVT: Invitro transcription.

**References**

Vogels, C. B. F., Brito, A. F., Wyllie, A. L., Fauver, J. R., Ott, I. M., Kalinich, C. C., et al. (2020a). Analytical sensitivity and efficiency comparisons of SARS-CoV-2 RT–qPCR primer–probe sets. Nat. Microbiol. doi:10.1038/s41564-020-0761-6.

Link to the Zang Lab protocol

https://www.broadinstitute.org/files/publications/special/COVID- 19%20detection%20(updated).pdf
